# Representative Survey for Evaluating Housing and Manure Handling Technologies of the Hungarian Pig Sector

**DOI:** 10.3390/ani13162658

**Published:** 2023-08-18

**Authors:** Zsuzsanna Benedek, Károly Dublecz, Ilona Anna Koltay, Gábor Fitos, Vanda Kisanna Várhelyi, Marianna Magyar, Béla Pirkó, Nóra Hegedűsné Baranyai

**Affiliations:** 1 Institute of Animal Husbandry Sciences, Georgikon Campus, Hungarian University of Agriculture and Life Sciences, 8360 Keszthely, Hungary; benedek.zsuzsanna@uni-mate.hu; 2 Institute of Physiology and Nutrition, Georgikon Campus, Hungarian University of Agriculture and Life Sciences, 8360 Keszthely, Hungary; dublecz.karoly@uni-mate.hu; 3 Association of Hungarian Pig Breeders and Keepers, 2053 Herceghalom, Hungary; ilcsu92@gmail.com (I.A.K.); fitos.gabor@mstsz.eu (G.F.); 4 Breeding Association for the Muraközi Horse, 9798 Ják, Hungary; vanda.k.varhelyi@gmail.com; 5 Centre for Agricultural Research, Institute for Soil Sciences, Department of Soil Chemistry and Material Turnover, 1022 Budapest, Hungary; pirko.bela@atk.hu; 6 Renewable Energy Research Group, University Center for Circular Economy, University of Pannonia Nagykanizsa, 8800 Nagykanizsa, Hungary; baranyai.nora@pen.uni-pannon.hu

**Keywords:** pig sector, age groups, housing, manure, ammonia mitigation

## Abstract

**Simple Summary:**

A major source of ammonia emissions in Hungary is pig production. The NEC Directive requires Hungary to achieve significant reductions in ammonia emissions by 2030 compared to the base year of 2005. In order to clarify the data available for 2005, a questionnaire was used to collect representative data on the housing and manure management technologies used in the Hungarian pig sector in 2005 and 2015, as well as the age groups and numbers of pigs affected by each technology. As our aim was to use the data for the calculation of the national emission inventory, a novel expert-based calculation method was developed to ensure that the data for the age group categories provided by the farmers were converted into the accepted statistical categories.

**Abstract:**

In Hungary, there is a lack of information on the pig production technologies in place in the base year of 2005 and changes since then, as well as a lack of information on the number of pigs kept in different age and production categories, which makes it difficult to calculate ammonia emissions and reductions in the national inventories. Our research team conducted a representative survey of pig farms to assess housing and manure management technologies in the Hungarian pig sector in 2005 and 2015. Novel expert-based calculation methods were developed to convert farm data on pig populations into daily average numbers (DAN) of animals in different statistical categories and feeding phases. The survey resulted in a representative database of housing, manure handling, storage and manure application practices in Hungarian pig production. The data and methodology from the survey helped to develop an ammonia emission calculator and knowledge transfer tool (AGEM-S) for use by farmers.

## 1. Introduction

The Paris Agreement [1], signed by 196 countries, including the EU, set the goal of achieving climate neutrality by 2050. Under international agreements, EU Member States are committed to reducing greenhouse gas emissions and air pollution [2,3,4]. In order to move towards levels of air quality that do not cause significant negative impacts and risks to human health and the environment, the NEC Directive and the revised Gothenburg Protocol [5] set emission reduction obligations for Member States from 2020 to 2029 and from 2030 onwards, taking 2005 as the base year. The NEC Directive requires Member States to develop, adopt and implement national air pollution control programmes and to monitor, document and report anthropogenic atmospheric emissions [4,6]. Hungary’s obligation under the NEC Directive is to reduce ammonia emissions by 10% in each year from 2020 to 2029 and by 32% in each year after 2030 compared to base 2005 emissions [4].

The largest emitting activity in terms of ammonia (NH_3_) and greenhouse gases is livestock production [7,8,9], and inadequate manure management in particular is a major contributor [10]. Agriculture, particularly intensive livestock production, is estimated to account for 50–85% of total US anthropogenic ammonia emissions [11], livestock manure is estimated to account for 40–49% of global NH_3_ emissions [12], and emissions are increasing in many countries and regions [13].

Ammonia is one of the most important atmospheric pollutants, damaging both natural ecosystems and human health [14,15,16]. Ammonia emissions occur at all stages of manure management. The quality and composition of the manure, as well as the way it is stored and handled, are the main factors determining the level of emissions from intensive livestock production [15,17]. The Hungarian Informative Inventory Report (IIRH) shows that, on trend, NH_3_ emissions from agriculture in Hungary will decrease by 44.6% between 1990 and 2021. One might think that this is a favourable reduction to meet the NEC requirements. However, the main driver of the reduction is the dramatic decrease in the number of pigs and cattle. The number of pigs in Hungary fell from 8 million in 1990 to 4.0217 million in 2005, with a further decline of 1.1845 million between 2005 and 2021. The problem is that the base year for the commitment to reduce ammonia emissions is 2005 and not 1990. The reduction in agricultural ammonia emissions was 44.6% between 1990 and 2021, but only 4.8% between 2005 and 2021 according to data available in 2023; although there was a 16.3% reduction between 2005 and 2012, the trend has since reversed. Based on 2021 data, the main source of ammonia emissions for all sectors was agriculture (92.1%), with the pig, poultry and cattle manure management sectors accounting for 44.1% of agricultural NH_3_ emissions [18].

Despite the extensive collection of statistical data on the pig population in Hungary, there is a lack of comprehensive information on the pig production technologies in place in the base year 2005 and changes since then, which causes difficulties in the calculation of ammonia emissions from the pig sector. The availability of data on the Hungarian pig population, disaggregated by age group and kept under different feeding, housing and manure management conditions, is the basis for accurate emission calculations. In order to obtain the most reliable data possible, our research team conducted a representative survey to evaluate the number of pigs from different feeding, housing and manure management technologies in the Hungarian pig sector for the years 2005 and 2015, which is presented in this paper. Assessing the changes that have been implemented over this ten-year period will allow understanding the direction and extent of them and help prepare for compliance with new environmental standards.

This data collection has provided the basis for the development of a decision support system to make properly based decisions on the technological improvements needed to reduce ammonia emissions. Such tools are already available to farmers in many countries around the world to help them understand the key drivers of ammonia emissions and plan effective mitigation measures. Examples include DATAMAN—emissions and emission factor databases [19,20,21,22]; the National Air Quality Site Assessment Tool [23]; the Air Management Practices Assessment Tool (AMPAT) [24,25]; the Feedlot Air Emissions Treatment Cost Calculator [26]; the Spreadsheet Decision Tools [27]; the Ammonia Emissions Estimator [28]; the Ammonia Losses from Liquid Manure Applications Calculator [29]; the Dairy Gas Emission Model [30]; SCAIL-Agriculture [31]; BAT-Tool Plus [32]; the Greenhouse Gas and Ammonia Emission Reduction Calculation Tool [33]; a calculation tool for ammonia emissions in the agricultural sector (SCENA) [34]; and Agrammon [35].

The developed system, called the Ammonia Gas Emission Model-Swine (AGEM-S) [36], is a farm-level knowledge transfer and decision support tool. The model calculates the number of animals in each feeding phase from the age and production group data provided by the farmers, and then estimates the annual nitrogen excretion of the animals.

## 2. Materials and Methods

### 2.1. Data Collection of the Hungarian Central Statistical Office

In Hungary, the number of animals in different livestock categories is collected by the Hungarian Central Statistical Office (HCSO). Hungary’s accession to the European Union resulted in the harmonization of the country’s statistical system. As of 2009, the HCSO conducts a livestock census twice a year (1 June and 1 December) in accordance with the new EU legislation, and the data are published 55 days after the census. The HCSO carries out routine revisions twice a year (in February and July) to verify the data. The categories of the pig population used by the HCSO are shown in Table 1 [37].

According to the HCSO, there were 3,853,000 pigs in Hungary in 2005. In 2005, 45.4% of the Hungarian pig population were fattening pigs over 50 kg, followed by growing pigs between 20 and 50 kg (22.4%), piglets under 20 kg (21.9%), breeding sows (7.2%), which included covered (or gestating) sows (4.8%) and sows not covered (2.4%), sows covered for the first time (1.4%), gilts not yet covered (1.4%), and boars (0.3%).

In Hungary, according to HCSO data, there were 3,124,000 pigs on 1 December 2015. In 2015, 45.5% of the Hungarian pig population were fattening pigs over 50 kg, followed by piglets under 20 kg (22.8%) and growers between 20 and 50 kg (22.7%), breeding sows (6.3%), gilts covered (4.5%), sows not covered (1.8%), gilts not yet covered (1.5%), sows covered for the first time (1.3%) and boars (0.2%).

### 2.2. Methods of Sampling and Data Collected

Over the course of the survey, a representative sample population was selected in order to determine the ammonia emission characteristics of the basic population of pigs kept on farms of different sizes and technological backgrounds. This estimation was based on the principles of probability calculation, so theoretically the selection of the sample itself could only be a random method.

The sampling method used was simple random sampling, where every pig farm in Hungary had an equal chance of being included in the sample. Equal chances meant that unintentional or intentional bias could be eliminated through predefined rules.

In the first round, 100 pig farms were randomly selected and contacted with the help of the Association of Hungarian Pig Breeders and Keepers. The farmers were informed in writing by the AHPBK about the aims of the research and data collection and the possible methods of data application, and the farmers were assured of complete anonymity.

Pig farms vary considerably in size. We also wanted to show the distribution of the pig population by herd size. Four farm sizes were included in the survey and we were able to obtain relevant information on household-, small-, medium- and large-scale farms. The farm sizes were defined as follows: household-scale farms with 0–3 sows, small-scale farms with 4–10 sows, medium-scale farms with 11–100 sows and large-scale farms with more than 100 sows. Farms with no sows were classified according to the number of other pigs that could be produced by the above number of sows. The number of data points collected from different farm sizes reflects the distribution of the population. Sampling was therefore carried out according to strict rules and it can be said that the data collection is unbiased in the representative population.

For data collection, a questionnaire in Excel format was prepared by the experts of the University of Pannonia, Georgikon Faculty, the Association of Hungarian Pig Breeders and Keepers and the National Agricultural Research and Innovation Centre, Institute of Agricultural Engineering. The form included questions in four main areas, such as general information on livestock and production, feeding and nutrition, housing technologies, and manure management for both 2005 and 2015.

In the first part, data on numbers and production were collected for the age and weight of different categories of animals (age or production groups) in the pig population. The genetic background of the herd, number of farrowings, average daily gain, length of periods (suckling, weaning, growing, fattening and rearing) and culling rates were also asked about. In other parts of the questionnaire, we asked for information on the average daily number of animals in different feeding phases or in different housing conditions. These figures had to be very precise in order to be able to quantify the distribution of different technologies and to estimate the ammonia emissions in the different facilities in later research. In order to achieve this goal, the questionnaire was designed on the principle that farmers should categorize their livestock data (daily average number of animals, DANA) according to their daily routine. In practice, the categories used by pig farmers correspond to the phases of housing technology.

For both years, the following age and production categories were used: gestating sows, lactating or nursing sows, weaner pigs or weaners, fattening pigs or fatteners, replacement gilts and boars. The age and production categories were divided into subgroups according to feeding phases.

The first part of the questionnaire also asked for some control data that could be used to prove the accuracy of the DANA reported by the respondents in the later parts of the questionnaire. For example: weaned piglets per sow per year, average daily gain and feed conversion ratio during growth and fattening.

Questions relating to housing and manure management technologies were asked for both 2005 and 2015, so that changes in technology between the two years could be identified.

For housing, the focus was on indoor and outdoor technologies, as these can have a major impact on ammonia emissions. Data were collected on populations kept indoors, i.e., in pig houses without paddocks, and on animals kept partly outdoors, i.e., in houses with paddocks.

In terms of manure management, liquid and solid manure techniques were surveyed. For liquid manure techniques, fully or partially slatted (or perforated) floors with slurry lagoons or pits underneath are relevant, and in Hungary the solid floor system without bedding can also be found. In solid manure management techniques, the solid floors of the pens are littered with bedding material to bind urine and faeces in the litter. There are two techniques relevant to solid manure management: the littered floor system and the deep litter system.

### 2.3. Methods of Analysis

During the processing of the completed questionnaires, some corrections and conversions were necessary. In some cases, the completed questionnaires were not complete enough and it was necessary to contact the respondents again by telephone. After this clarification, a total of 87 well-completed questionnaires could be used. The sampled number of pigs covered 20% of the represented base population for 2015.

#### 2.3.1. Calculating DAN of Nursing and Gestating Sows, Weaners and Fatteners

Using the livestock production data, the following expert calculation methods were used to verify and accurately determine the daily average number (DAN) of animals in different categories. DAN is the key figure that is also widely used in subsequent calculations.

(1) $$Farrowing\;rotation=\frac{365}{days\;of\;nursing}$$

(2) $$DAN\;of\;nursing\;sows=\frac{annual\;number\;of\;farrowings}{farrowing\;rotation}$$

(3) DAN of gestating sows=total number of sows−number of nursing sows

(4) Total number of replacement gilts=total number of sows ∗ culling rate of sows ∗ 1.5

(5) $$Weaner\;rotation=\frac{365}{days\;of\;weaner\;period}$$

(6) $$DAN\;of\;weaners=\frac{total\;number\;of\;weaners}{weaner\;rotation}$$

(7) $$Fattener\;rotation=\frac{365}{Days\;of\;Fattening\;Period}$$

(8) $$DAN\;of\;fatteners=\frac{total\;number\;of\;fatteners}{fattener\;rotation}$$

#### 2.3.2. Conversion of Farm Data on Pig Populations into Statistical Category Data

**Figure 1:**
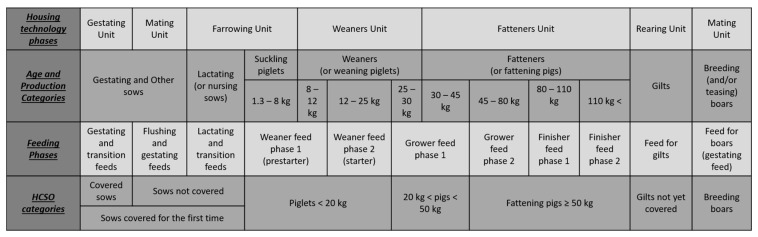
Different categorizations used for pig populations.

The questionnaire was designed to ensure that the information obtained from the representative sample could also be used by the Hungarian Meteorological Service (HMS) for the calculation of IIRH and by the Ministry of Agriculture (MA). As mentioned above, in order to obtain accurate data, farmers should classify their DAN of livestock into categories that are used in their daily routine. In practice, the age and production categories used by pig farmers correspond mainly to the housing technology phases and partly to the feeding phases, but this is not the case at all for the HCSO categories. Figure 1 shows the different categorizations used for pig populations.

It should be noted that the age and production categories for pigs used in official statistics may need to be revised as far as possible at international level, as they do not correspond to the official nomenclature of the pig farming sector and there is therefore a great potential for misunderstanding or incorrect data collection.

This is particularly the case when recording the number of different categories of sows. In pig farming practice, breeding sows are not recorded as ‘sows not covered’, which means sows under mating and in lactation together in the statistical nomenclature. The reason for this is that sows under mating (which can also be referred to as other sows, as sows mated but not in gestation are also included in this group) are housed separately from lactating sows in mating or gestating stalls, while the lactating sows are housed in the farrowing units, as it can be seen in Figure 1. The technology of these two types of housing is completely different, and these production categories also require completely different feeding regimes. Consequently, these groups cannot be treated together in terms of emissions either. These facts also support the need to revise the statistical data collection for age and production groups in the pig sector.

However, not only production, but also age categories had to be adjusted, as shown earlier in Figure 1.

As the calculations in the IIRH are based on the HCSO livestock category figures, it was necessary to convert the age and production groups of the questionnaire into the categories used by the HCSO in order to use them for the IIRH calculations.

During the survey, our research group developed a novel method to convert farm data on pig populations in different categories into the data of the statistical categories. The calculation method uses partition coefficients. In fact, the partition coefficient of an age or production category is based on the ratio of the time the animals spend in a group compared to the length of a longer production phase. First, the length (in days) of the different age or production categories must be known or calculated. Then, the known DANA in the longer production phase should be multiplied by the division coefficient of the statistical category concerned to obtain its DAN of livestock.

##### Conversion of DAN of Weaners and Finishers to DAN of Piglets < 20 kg, DAN of 20 kg < Pigs < 50 kg and DAN of Finishers ≥ 50 kg

(9) $$Rearing\;days\;of\;piglets<20\;kg=\frac{20\;kg\;-\;weaning\;weight}{average\;daily\;gain\;of\;weaning\;period}$$

(10) $$Rearing\;days\;of\;20\;kg\;<\;pigs\;<\;50\;kg\;=\;\frac{50\;kg-20\;kg}{average\;daily\;gain\;of\;weaning\;and\;fattening\;period}$$

(11) $$Rearing\;days\;of\;fattening\;pigs\;\geq \;50\;kg\;=\;\frac{finishing\;weight\;-\;50\;kg}{average\;daily\;gain\;of\;fattening\;period}$$

The ratio of the rearing days of the three age groups to the total rearing and finishing period (days from weaning to slaughter) gives the partition coefficient for each statistical category. Multiplying the total DAN of weaners and fatteners by the partition coefficients gives the DAN of the statistical categories.

##### Calculation of DAN for the Different Statistical Categories of Breeding Sows, Such as First-Time, Non-Covered and Covered Sows

In practice, the DAN category of lactating sows must be divided into sows in first lactation and sows not covered, as sows in first lactation belong to the statistical group of sows covered for the first time, while lactating sows belong to the statistical group of sows not covered:

(12) Sows in first lactation = Lactating sows ∗ culling rate of sows

(13) Sows not covered = Lactating sows − sows in first lactation

The DAN of gestating and other sows must be divided into sows in first gestation and covered sows, as these are statistically separate categories:

(14) Sows in first gestation = Gestating and other sows ∗ culling rate of sows

(15) Covered sows = Gestating and other sows − sows in first gestation

The DAN of sows covered for the first time is the sum of sows in first gestation and first lactation.

(16) Sows covered for the first time = Sows in first lactation + Sows in first gestation

The DAN of livestock in other statistical categories is the same as that used in practice. The detailed conversions make it possible to present the results of the questionnaire in accordance with statistical expectations.

#### 2.3.3. Calculation of the Annual Number and DAN for the Additional Feeding Categories of AGEM-S Based on the Input Data Provided by the Farmers

The production categories distinguished in pig farming practice do not always correspond to the feeding phases, and several feeding phases can typically be introduced in one age group in order to adapt the feed content of the diet as closely as possible to the physiological needs of the animal, thus increasing the incorporation rate and reducing the environmental impact (Figure 1).

The feeding module of AGEM-S is designed to calculate, in the background, the DANA from the data provided by the farmers for the age group categories, and from DANA the N excretion and the TAN values relevant for ammonia emissions by incorporating correction factors for each ammonia-reducing feeding method. The amount of N excreted is the input to the AGEM-S farm technology module.

(17) $$Replacement\;gilts\;rotation\;=\;\frac{365}{days\;of\;replacement\;gilts\;period}$$

(18) $$DAN\;of\;replacement\;gilts=\frac{Total\;number\;of\;replacement\;gilts}{days\;of\;replacement\;gilts\;period}$$

(19) Total number of weaner=annual number of farrowings ∗ number of litters at farrowing−mortality at farrowing unit

(20) $$DAN\;of\;weaners=\frac{total\;number\;of\;weaners}{weaner\;rotation}-\;half\;of\;the\;mortality\;at\;weaners\;unit$$

(21) Total number of growers=total number of weaners−total number of sold weaners − mortality at weaners unit 

(22) Total number of sold fattener = Total number of growers − mortality at fattener unit

(23) $$DAN\;of\;fatteners=\frac{total\;number\;of\;growers}{fattener\;rotation}-half\;of\;the\;total\;mortality\;at\;fattening\;unit$$

## 3. Results

According to the HCSO, there were 3.853 million pigs in Hungary in 2005. During the survey, information was collected from 58 farms that were already operating in 2005. In total, data were provided for 385,213 pigs, which is 10% of the total number of pigs in Hungary, referring to the base year for the reduction of ammonia emissions.

### 3.1. Swine Population by Age and Production Categories

In 2005, the proportion of age groups and production categories in the sample population was similar to that in the base population for the 58 holdings. Again, fattening pigs predominated, with a proportion of 42.2%.

In 2015, the database of the assay included 600,444 pigs on 86 farms, representing 19.2% of the total pig population. The sampling rate in the base population was above 10% in all age groups except for the boar rate, and the distribution was 49.8% in the sows covered for the first time. Considering the 86 farms, the distribution of age groups in the sample was similar to that in the base population. Fattening pigs over 50 kg also dominated, as in the base population. However, as in 2005, the sample was followed by the growers between 20 and 50 kg, followed by piglets under 20 kg, in contrast to the base population, where the size of these two age groups was quite similar. This can be explained by the fact that HCSO also includes suckling piglets in the age group of piglets under 20 kg. In this case, the period lengths of the piglets and growers are nearly the same in the two HCSO age groups, if the typical weight gains of the groups are considered. From the point of view of ammonia emission, the suckling piglets are not worth examining, while their feed is almost 100% sow’s milk, at least until the 21st day of life. The feed intake of suckling piglets in the form of milk is in included in the sows’ feed. The sows’ nitrogen retention also includes the piglets’ milk nitrogen intake. The manure after suckling piglets is relatively small and cannot be treated separately from that of the lactating sows. Therefore, considering the suckling piglets separately in ammonia emission calculations can lead to distortion. In the sample population, the rate of these two age groups is calculated from the time of weaning, and therefore truly represents the length of the weaning and growing periods.

On these 58 farms—which were already in operation in 2005—the herd increased in size by 10.3% by 2015 (from 385,213 pigs to 424,790 pigs). Furthermore, the distribution of age groups in the samples showed similarities in both years. Fattening pigs over 50 kg accounted for the largest proportion, with more than 42% of the total, which is not surprising as this is the longest of the age groups.

One of the key areas of the survey was to establish what type of housing technology was being used in 2005 and whether there had been any progress over the ten years. This is of interest not only for the farms but also for the age and size groups. The results are shown in Table 2 below.

### 3.2. Housing Technology

In both 2005 and 2015, the closed housing system without paddocks dominated in all age groups, but in different proportions. On the farms that were still in operation in both 2005 and 2015, the use of stalls with paddocks decreased in all groups. In relation to the total population, there was a 17.2% increase in the number of pigs kept in closed systems (this is a real increase and not just the result of a 10.3% increase in the population). At the same time, the number of animals kept in paddocks decreased by 40%, which means that 18,770 fewer pigs were kept outdoors for part of the time. The increase in the DAN of pigs kept indoors can be seen as a measure to reduce ammonia emissions.

In 2005, 88.1% of the sample population, including 99.4% of piglets under 20 kg, 96.1% of growers between 20 and 50 kg, 90.7% of sows not covered, 88.4% of fatteners over 50 kg, 59.3% of boars, and 53.7% of sows covered for the first time were housed in closed buildings. However, stalls with paddocks were typical for 55% of gestating sows and common for gilts. Overall, the age groups with the highest manure production (weaners and finishers) were typically housed indoors. Housing pigs outdoors was typical only for age groups whose size was not so significant in the total population (6% of the total).

In 2015, 94.5% of the sample population was predominantly housed indoors: 94.5% of piglets under 20 kg, 98.6% of growers between 20 and 50 kg, 97.5% of sows not covered, 94.2% of fattening pigs over 50 kg, 84.5% of boars, 79.4% of sows covered for the first time, 75.1% of gestating sows, and 72.1% of gilts not yet covered. Stalls with paddocks decreased in all age groups. In terms of population, the number of indoor stocks increased by 6.3%. The main increase occurred in the groups of gestating sows (72.4%), and gilts not yet covered (58.5%), as the technological trends changed over the ten years.

In 2005, in addition to fattening pigs, most of the pigs kept in stalls with paddocks belonged to the groups of covered sows (19%), sows covered for the first time (14.6%) and gilts not yet covered (13.9%). The reason for the above distribution can be explained by the beneficial effects of sunlight and exercise on the breeding stock, such as the improvement of constitutional and reproductive traits.

### 3.3. Farm Size

In 2005, of the 58 farms surveyed, 3 were household farms, 5 were small-scale farms, 23 were medium-scale farms and 27 were large-scale farms. During this period, 291,853 pigs were kept on large-scale farms, 90,719 pigs on medium-scale farms, 2500 pigs on small-scale farms and 140 pigs on household farms in the sample.

In 2015, considering all the farms surveyed, 7 were household farms, 6 were small farms, 28 were medium farms and 45 were large farms. During the ten years there was a noticeable reorganization among the farm size categories. Of the farms that were active in both 2005 and 2015, one small-scale farm became a medium-scale farm and four medium-scale farms became large-scale farms. In addition, one large-scale holding significantly reduced its number of animals and became a household holding.

Of the total number of pigs monitored in the sample, 491,435 pigs were kept on large-scale farms, 104,564 pigs on medium-scale farms, 3007 pigs on small-scale farms and 215 pigs on household farms in 2015. Among the farms that were also in operation in 2005, there were 344,647 pigs in large-scale farms, 77,543 pigs in medium-scale farms, 2156 pigs in small-scale farms and 143 pigs in household farms.

Table 3 and Table 4 show how the pig population changed between 2005 and 2015 according to age and production groups and farm size categories. It can be seen that in 2005, the majority of the household stock was kept in stalls with paddocks, while the other three categories were generally kept indoors (Table 3). In 2015, 92% of household livestock and 51.3% of small-scale livestock were kept in stalls with outdoor technology. In total, 86.1% of medium-scale farms and 96.9% of large-scale farms used only indoor housing (Table 4). In addition, outdoor housing was predominant on household farms for piglets, growers, fattening pigs and gilts, while sows and boars were typically kept indoors. In small holdings, piglets, sows and boars were also kept indoors, while in medium and large holdings all animals were kept indoors.

**Figure 2:**
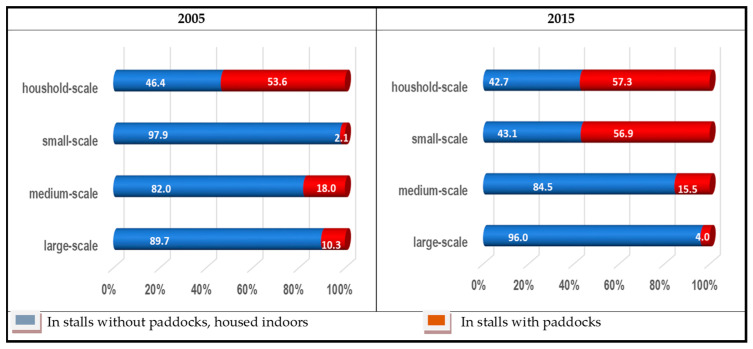
Distribution of housing types on farms of different sizes in 2005 and 2015, %. Source: according to the survey.

Looking at those holdings that were also active in both years (Figure 2), it can be seen that in 2005 only household farms kept more than half of their livestock outdoors. In 2015, both household and small-scale holdings used this technique, while the other two types of holdings increased their indoor housing by 3% and 7.4%, respectively.

### 3.4. Manure Management Techniques

According to the survey, it must be taken into account that not all farms have information on manure management techniques. In 2005, information on manure management systems was provided for 371,567 pigs, while in 2015, 559,477 pigs were included in the sample.

In 2005, the highest number of animals (DANA was 92,894 pigs) of the represented population were kept on partially slatted floors over lagoon systems with less than one month of slurry storage time. In 2015, fully slatted floors over slurry pits with more than one month of storage time was the most popular of all manure handling technologies (DANA was 146,866 pigs).

Looking at the farms that were in operation in both 2005 and 2015, the DANA decreased for the following manure management systems: the littered floor system by 26.5%, deep litter (more than one month of storage) by 5.8%, fully slatted floor with lagoons by 28.4%, partly slatted floor with lagoons by 22.9%, and solid floor without bedding by 4%, respectively. There was a detectable increase in the rate of the following systems: deep litter with less than a month of manure storage (12.1%), fully slatted floor with lagoons and less than a month of slurry storage (104.6%), and fully slatted floor with slurry pit and more than one month of slurry storage (127.2%). The most significant increase was seen in the categories of fattening pigs over 50 kg and covered sows, where fully slatted floor with slurry pits for manure handling increased by 406.6%, and 410.3%, respectively.

Table 5 shows the distribution of manure management techniques according to the age groups of the pig population in 2005. In each group, bedded housing was the most popular technique for boars (51.6%), fully slatted floors for piglets (25.6%) and sows not covered (20.1%), and partially slatted floors for fattening pigs (40.6%). In the groups of growers (18%), gestating sows (39.4%), sows covered for the first time (38.2%) and gilts (44.4%), solid floors without bedding were used most frequently.

By 2015, the technology had changed both in terms of age and production groups and complexity, as shown in Table 6. Whereas in 2005 a quarter of the population was housed on partially slatted floors with lagoon systems, ten years later fully slatted floors with slurry pits and more than one month of manure storage were dominant (26.3%). A reorganization can also be seen in the manure handling of the different age groups, but in the case of boars, solid manure is still the primary approach (38.8%).

As AGEM-S was designed with the aim of creating a model adapted to Hungarian conditions, knowledge of the technologies that are widely used in practice was a basic requirement. The development of AGEM-S was therefore based on this pig technology survey, which resulted in a representative database of the housing, manure handling, storage and manure application methods used in Hungarian pig production.

## 4. Discussion

The aim of the livestock and technology survey carried out was to collect detailed data that can be used to clarify the feeding, housing and manure management technologies used by farms and the related animal numbers. The data will provide a country-wide representative overview of the trends in livestock numbers and the proportions of ammonia non-compliant, compliant and modern technologies. Equally important is the fact that the comparison of the two time periods provides an opportunity to analyse the changes and identify the direction of modernization to date.

It is important to note, however, that the survey data cannot be compared with other surveys in Hungary, as they are not available. Comparison with practices in other countries is also limited due to the different structure of data collection. The new methodology described in this study, which will help to compare data collection with different structures, and may even help at the EU level in the future, will be used to calculate and match data from different sources for age and utilization groups and feeding stages.

In Hungary, the overall pig population decreased in the period under study, but the sector is characterized by concentration, with a shift towards larger pig farms. We found that in both 2005 and 2015, the number of pigs kept without paddocks increased by 17.2%. Animal welfare reasons [38] would justify the maintenance of paddocks, especially for breeding animals and the rearing of breeding piglets, while animal health and disease control restrictions [39] favour the spread of confined housing. Our investigations confirmed that, in line with international trends, the proportion of pigs kept in large-scale pig farms has increased in Hungary and the number of household- and small-scale pig farms has decreased, i.e., concentration is prevalent in the sector, while the total pig population is decreasing. This phenomenon may also have played a role in the decrease in the proportion of stalls with paddocks, as this technology is rarely used on large farms.

There was a shift in manure management technologies during the period under review. While in 2005 the most common bedding technology used on farms was partly slatted floors with a storage period of less than one month, by 2015 fully slatted floor technology had become the most common, with a storage period of more than one month. While this is a negative development in terms of ammonia emissions, the longer storage time makes it easier for farms to organize manure spreading.

In the years following EU accession, between 2007 and 2012, the drivers for technological change were the four application programmes for the modernization of livestock farms under the Common Agricultural Policy. The aim of these was to bring livestock farms in line with the standards of manure spreading, and to improve the applied feed and technical quality, the animal health and food safety, and the working conditions and efficiency of livestock farm workers. Nevertheless, there has been little independent initiative in the field of slurry management and utilization. Subsequent changes have come about under pressure from the EU’s strict environmental standards.

In Hungary, the Nitrate Directive came into force in 2002, which gradually tightened the conditions for manure storage, and the length of the application ban period increased significantly during the period under study. This has led to a shift towards longer storage technology. This has brought farms closer to compliance with the Nitrate Directive, but further away from the air quality standards that came into force later. In our view, this is a good example of the pressure on farmers from constantly strengthening and sometimes conflicting environmental regulations.

In recent decades, international research has identified sources of ammonia emissions and developed technologies to reduce emissions. At the same time, there is a growing recognition that knowledge transfer and decision support tools can provide farmers with a wide range of practical support for the use of existing knowledge. At the time of our study, however, there were no freely available tools in Hungary that could support farmers in developing environmental thinking or complying with environmental regulations through knowledge transfer. By developing AGEM-S, we aimed to fill this gap.

Several international models (e.g., Agrammon [35]; MAM [40]; NEMA [41]; NARSES [42,43]; and DNDC Manure [44]) were studied for adaptability before AGEM-S was developed. These models have in common that they use a large amount of input data in the calculation process, which are often difficult for farmers to access. In our view, data requirements covering too many parameters undermine the willingness to provide data even if the data are otherwise available in one of the registers. For these reasons, our own efforts were aimed at developing a farmer-oriented decision support tool by transforming the input data in a practice-driven way without compromising the accuracy of the model.

For this purpose, the requested input data are based, without exception, on information known on a daily basis by farmers. This makes the input parameters as simple as possible to enter and can be achieved in the shortest possible period of time, thus increasing the willingness to use the calculator. The novelty of our development lies in the fact that farmers only need to enter the age group data and reproductive and production characteristics used on a daily basis, which are used by the calculations in the background to provide accurate livestock data for estimating the annual on-farm N and TAN excretion and thus ammonia emissions.

## 5. Conclusions

Our research allows us to evaluate the housing and manure management technologies of the Hungarian pig sector for the years 2005 and 2015. Detailed and precise data are presented on the average daily number of pigs in different age and production groups kept under different housing and manure management conditions.

During the survey, our research group developed a new expert-based calculation method for converting farm data on pig numbers.

This novel calculation method can be used throughout the EU to convert farm data into different types of statistical data regarding age and population groups.

Knowing the distribution of statistical categories of pigs kept in different technologies with different emissions allows us to clarify the calculation of ammonia emissions for the two years studied and to evaluate the changes in ammonia emissions over the ten-year period.

Raising awareness about reducing emissions is an important task, and the results of representative surveys, knowledge transfer, and decision support tools such as AGEM-S are essential.

## Figures and Tables

**Table 1:** Statistical data collection categories for pigs [37].

Pig Population Categories	Attributes
Breeding sows	Covered sows + cows not covered (mating and lactating sows)
Breeding pigs	Breeding sows + boars
Piglets	≤19 kg
Pigs	20–49 kg
Fattening pigs	≥50 kg
Fattening pigs I	50–79 kg
Fattening pigs II	80–110 kg
Fattening pigs III	≥110
Breeding boars	
Sows covered for the first time	
Gilts not yet covered	

**Table 2:** The changes in numbers of swine (DANA) according to housing in each age and production category in 2005 and 2015.

Category	Swine in Stalls, without Paddocks, in Closed Buildings		Stalls with Paddocks	
	2005	2015	2005	2015
Piglets under 20 kg	65,195	93,171	383	250
Growers, 20–50 kg	106,338	167,333	4291	2378
Fattening pigs, over 50 kg	143,763	256,410	18,841	15,730
Covered sows	7109	15,525	8688	5157
Sows not covered	3963	6392	406	162
Sows covered for the first time	7741	15,821	6684	4113
Gilts not yet covered	5146	12,721	6358	4933
Boars	181	294	124	64
Total	339,437	567,666	45,776	32,787

Source: own calculation, according to the questionnaire.

**Table 3:** Distribution of the swine population (DANA) in different age and production groups and farm size categories according to housing systems in 2005 (%). Source: according to the survey.

Category	Piglet, under 20 kg	Growers, 20–50 kg	Fattening Pig, over 50 kg	Covered Sow	Sows Not Covered	Sows Covered for the First Time	Gilt	Boar	All Together
Household-scale farms									
In stalls, without paddocks, housed indoors	0.0	0.0	23.5	15.1	100.0	n.a	n.a	100.0	46.4
In stalls with paddocks	100.0	100.0	76.5	84.9	0.0	n.a	n.a	0.0	53.6
All together	100.0	100.0	100.0	100.0	100.0	n.a	n.a	100.0	100.0
Small-scale farms									
In stalls, without paddocks, housed indoors	100.0	100.0	100.0	40.4	100.0	54.8	52.0	61.5	94.7
In stalls with paddocks	0.0	0.0	0.0	59.8	0.0	46.0	48.0	38.5	5.3
All together	100.0	100.0	100.0	100.0	100.0	100.0	100.0	100.0	100.0
Medium-scale farms									
In stalls, without paddocks, housed indoors	97.5	93.3	74.9	52.8	91.8	66.9	65.8	65.6	82.8
In stalls with paddocks	2.5	6.7	25.1	47.2	8.2	33.2	34.2	34.4	17.2
All together	100.0	100.0	100.0	100.0	100.0	100.0	100.0	100.0	100.0
Large-scale farms									
In stalls, without paddocks, housed indoors	100.0	97.0	92.9	43.2	90.3	50.8	39.1	43.9	89.7
In stalls with paddocks	0.0	3.0	7.1	56.8	9.7	49.2	60.9	56.1	10.3
All together	100.0	100.0	100.0	100.0	100.0	100.0	100.0	100.0	100.0

**Table 4:** Distribution of the swine population (DANA) in different age and production groups and farm size categories according to housing systems in 2015 (%). Source: according to the survey.

Category	Piglet, under 20 kg	Growers, 20–50 kg	Fattening Pig, over 50 kg	Covered Sow	Sows Not Covered	Sows Covered for the First Time	Gilt	Boar	All Together
Household-scale farms									
In stalls, without paddocks, housed indoors	0.0	47.3	48.2	23.6	100.0	100.0	0.0	90.2	8.0
In stalls with paddocks	100.0	52.7	51.8	76.4	0.0	0.0	100.0	9.8	92.0
All together	100.0	100.0	100.0	100.0	100.0	100.0	100.0	100.0	100.0
Small-scale farms									
In stalls, without paddocks, housed indoors	80.0	40.8	49.4	37.7	89.3	55.3	23.5	57.1	48.7
In stalls with paddocks	20.0	59.2	50.6	62.3	10.7	44.7	76.5	42.9	51.3
All together	100.0	100.0	100.0	100.0	100.0	100.0	100.0	100.0	100.0
Medium-scale farms									
In stalls, without paddocks, housed indoors	98.8	96.1	77.6	71.7	100.0	82.3	74.5	98.2	86.1
In stalls with paddocks	1.2	3.9	22.4	28.3	0.0	17.7	25.5	1.8	13.9
All together	100.0	100.0	100.0	100.0	100.0	100.0	100.0	100.0	100.0
Large-scale farms									
In stalls, without paddocks, housed indoors	100.0	99.6	98.2	76.2	97.0	79.0	78.3	75.5	96.9
In stalls with paddocks	0.0	0.4	1.8	23.8	3.0	21.0	21.7	30.7	3.1
All together	100.0	100.0	100.0	100.0	100.0	100.0	100.0	100.0	100.0

**Table 5:** The share of manure management systems in different age and production groups, 2005 (%). Source: according to the survey.

Category	Piglet, under 20 kg	Growers, 20–50 kg	Fattening Pig, over 50 kg	Covered Sow	Sows Not Covered	Sows Covered for the First Time	Gilt	Boar	All Together
Littered floor system. Remove manure daily	10.8	17.0	26.8	28.5	15.1	23.3	19.0	51.6	20.8
Deep litter. Remove manure in less than month intervals	0.0	0.5	0.4	0.6	0.0	0.2	0.1	1.6	0.3
Deep litter. Remove manure in more than month intervals	14.3	6.3	4.0	4.0	5.0	2.4	0.5	5.8	6.1
Fully slatted floor with lagoons underneath and less than a month of manure storage	11.0	10.0	4.9	0.9	18.4	6.3	10.1	1.9	7.7
Fully slatted floor with slurry pits underneath and more than a month of manure storage	16.8	14.4	3.1	1.3	5.6	2.7	15.4	1.9	9.2
Fully slatted floor with other techniques	25.6	13.2	0.7	1.6	20.1	6.9	0.0	0.0	8.8
Partly slatted floor with lagoons underneath and less than a month of storage	4.9	17.9	40.6	23.0	16.0	19.8	10.1	5.8	25.0
Solid floor without bedding and with channels for slurry transfer	15.0	18.0	16.4	39.4	19.8	38.2	44.4	29.5	19.7
Further techniques	1.7	2.8	3.1	0.7	0.0	0.3	0.3	1.9	2.4
All together	100.0	100.0	100.0	100.0	100.0	100.0	100.0	100.0	100.0

**Table 6:** The share of manure management systems in different age and production groups, 2015 (%). Source: according to the survey.

Category	Piglet, under 20 kg	Growers, 20–50 kg	Fattening Pig, over 50 kg	Covered Sow	Sows Not Covered	Sows Covered for the First Time	Gilt	Boar	All Together
Littered floor system. Remove manure daily	14.0	8.2	12.9	17.0	11.5	15.7	18.9	38.8	12.1
Deep litter. Remove manure in less than month intervals	0.0	0.3	0.2	0.8	0.0	0.4	0.3	2.2	0.2
Deep litter. Remove manure in more than month intervals	2.3	5.3	16.1	1.8	0.0	3.6	0.1	8.1	9.4
Fully slatted floor with lagoons underneath and less than a month of manure storage	27.4	16.6	7.6	6.3	31.8	12.8	6.5	2.6	13.7
Fully slatted floor with slurry pits underneath and more than a month of manure storage	34.6	37.7	19.0	12.6	14.0	15.8	11.8	16.5	26.3
Fully slatted floor with other techniques	9.2	5.3	1.8	2.7	24.4	8.9	0.9	0.0	4.5
Partly slatted floor with lagoons underneath and less than a month of storage	7.9	15.2	25.7	28.2	10.8	22.1	16.5	11.4	19.4
Solid floor without bedding and with channels for slurry transfer	4.5	10.6	14.7	30.3	7.4	20.5	37.3	16.1	13.2
Further techniques	0.0	0.7	2.0	0.5	0.0	0.3	7.7	4.4	1.3
All together	100.0	100.0	100.0	100.0	100.0	100.0	100.0	100.0	100.0

## Data Availability

Not applicable.

## Abbreviations

AGEM-S	Ammonia Gas Emission Model for Swine (in Hungarian), https://agem.naik.hu/ (accessed on 15 August 2023)
AMPAT	Air Management Practices Assessment Tool
DAN	Daily average number
DANA	Daily average number of animals
DATAMAN	Database of Greenhouse Gas Emissions from Manure Management, https://www.dataman.co.nz (accessed on 15 August 2023)
EU	European Union
HCSO	Hungarian Central Statistical Office
HMS	Hungarian Meteorological Service
IIRH	Hungarian Informative Inventory Report
MA	Ministry of Agriculture
NEC Directive	Directive on national emission ceilings for certain atmospheric pollutants
NH_3_	Ammonia
